# Chemotherapeutic effects on breast tumor hemodynamics revealed by eigenspectra multispectral optoacoustic tomography (eMSOT)

**DOI:** 10.7150/thno.56173

**Published:** 2021-06-26

**Authors:** Evangelos Liapis, Angelos Karlas, Uwe Klemm, Vasilis Ntziachristos

**Affiliations:** 1 Helmholtz Zentrum München (GmbH), Neuherberg, Germany, Institute of Biological and Medical Imaging.; 2 Technical University of Munich, Germany, School of Medicine, Center for Translational Cancer Research (TranslaTUM), Chair of Biological Imaging.

**Keywords:** optoacoustic, photoacoustic, breast cancer, docetaxel, Taxotere, chemotherapy

## Abstract

Non-invasive monitoring of hemodynamic tumor responses to chemotherapy could provide unique insights into the development of therapeutic resistance and inform therapeutic decision-making in the clinic.

**Methods:** Here, we examined the longitudinal and dynamic effects of the common chemotherapeutic drug Taxotere on breast tumor (KPL-4) blood volume and oxygen saturation using eigenspectra multispectral optoacoustic tomography (eMSOT) imaging over a period of 41 days. Tumor vascular function was assessed by dynamic oxygen-enhanced eMSOT (OE-eMSOT). The obtained *in vivo* optoacoustic data were thoroughly validated by *ex vivo* cryoimaging and immunohistochemical staining against markers of vascularity and hypoxia.

**Results:** We provide the first preclinical evidence that prolonged treatment with Taxotere causes a significant drop in mean whole tumor oxygenation. Furthermore, application of OE-eMSOT showed a diminished vascular response in Taxotere-treated tumors and revealed the presence of static blood pools, indicating increased vascular permeability.

**Conclusion:** Our work has important translational implications and supports the feasibility of eMSOT imaging for non-invasive assessment of tumor microenvironmental responses to chemotherapy.

## Introduction

The ability to spatially map tumor hypoxia on a global scale within solid tumors could offer valuable insights into the extent of tumor heterogeneity and ultimately facilitate cancer staging and prognosis. Moreover, non-invasive, longitudinal tracking of hemodynamic and oxygenation changes in tumors could potentially improve the clinical outcome in patients undergoing chemotherapy by enabling rapid assessment of treatment response as well as optimization of dosing and scheduling with combined radiotherapy or other treatments. For these reasons, there is an urgent need to develop non-invasive imaging biomarkers that measure the extent and spatial distribution of hypoxia in tumors and track changes in oxygenation and vascular function in response to therapy.

Docetaxel (Taxotere^®^), is an anti-neoplastic drug of the taxoid family and is a semi-synthetic analogue of paclitaxel 1, 2. Its primary molecular mechanism of action is hyperstabilisation of microtubules 3 which leads to mitotic cell arrest and eventually cell death by apoptosis 4-6. In addition, it can induce apoptosis through the stimulation of phosphorylation of bcl-2 2, 5-7. Docetaxel has emerged as one of the most active chemotherapeutic drugs available for the treatment of patients with locally advanced or metastatic breast cancer after failure of prior chemotherapy (for reviews, see 5, 6, 8-10) and has shown to significantly improve pathologic response rates in the neoadjuvant breast cancer setting 11, 12. Despite the clinical benefits of docetaxel treatment, not all patients respond and eventually almost all patients develop tumors that are non-responsive to this drug due to the emergence of resistance through multiple biochemical pathways 13. In addition to genetically altered characteristics of cancer cells, it is increasingly recognized that the tumor microenvironment (TME) plays a key role in cancer progression and in determining the therapeutic response to different chemotherapy regimens 14-17, including taxanes 18, 19. In particular, the abnormal vasculature of solid tumors reduces vascular perfusion, leading to diminished delivery and penetration of drugs to tumor sites 20 and, importantly, to the development of acidic and hypoxic areas 21. The presence of such hypoxic regions in solid tumors is strongly associated with increased tumor aggressiveness and is a negative prognostic factor for survival and local control in cancer patients, independent of the clinical stage at diagnosis 22-24. Furthermore, mounting studies suggest that tumor hypoxia limits the efficacy of chemotherapy and confers resistance to taxanes and other anti-proliferating agents through numerous cellular pathways, such as induction of cell quiescence and evasion of apoptosis 18, 19.

Continuous monitoring of the dynamic changes in TME over the course of taxane treatment would thus have wide implications in the preclinical development of taxane drugs as well as in clinical decision making and personalized medicine. However, assessment of tumor hypoxia is currently challenging, primarily due to its heterogeneous and dynamic nature and also the absence of non-invasive imaging methods capable of reporting the oxygenation status of tumors accurately and repeatedly at high spatiotemporal resolution and over extended periods of time. Existing clinical imaging modalities such as positron emission tomography (PET) and several magnetic resonance imaging (MRI) based techniques have key limitations, such as low spatial and temporal resolution and high cost, and additionally require the injection of chemical contrast agents or radiotracers which suffer from poor delivery to and uptake by tumor cells due to the highly abnormal tumor vasculature 21, 25.

Recently, optoacoustic imaging has been heralded as an emerging non-invasive technique that offers the prospect of overcoming the limitations of the aforementioned methods. In particular, a 3D optoacoustic technique known as multispectral optoacoustic tomography (MSOT) provides robust, real-time assessment of vascular function and blood oxygenation at several centimeters of tissue depth, in addition to anatomical information, by acquiring data at multiple wavelengths 26. To date, the technique has been applied in various preclinical therapeutic studies of engrafted tumors in mice 27-32 and is currently in clinical trials 33-35. Nonetheless, the dependence of optical fluence on tissue composition, depth and wavelength (an effect known as "spectral coloring”) poses a major challenge to spectral unmixing and confounds quantification of hemoglobin oxygen saturation (sO_2_), especially with increasing imaging depth. A recently developed technique in our lab known as eigenspectra multispectral optoacoustic tomography (eMSOT) was shown to substantially improve quantification of oxygenation over conventional linear unmixing methods in proof-of-concept *in vitro* and *in vivo* experiments 36, 37 and to correlate with immunohistochemistry in animal breast tumor models 32. Furthermore, we have previously demonstrated the potential of the method in resolving tumor oxygenation with high spatial resolution and sensitivity over the course of bevacizumab treatment in breast xenografts 32.

In the present study, we set out to delineate the effects of Taxotere monotherapy on breast tumor hemodynamics and oxygenation in a longitudinal manner using eMSOT imaging. To achieve this, we used an orthotopic HER2-positive xenograft model of advanced inflammatory breast cancer (KPL-4) with established responsiveness to docetaxel 38. Moreover, in order to gain a better insight into the vascular function of Taxotere-treated KPL-4 tumors, we applied an oxygen challenge as similarly reported in previous optoacoustic studies 27, 39, 40. Our results support the preclinical application of eMSOT imaging to monitor hemodynamic responses to chemotherapy and provide unique insights into the modifying effects of Taxotere on the vascular TME.

## Material and Methods

### Study design

The research objective of our study was to determine the effects of continuous Taxotere monotherapy on breast tumor oxygenation and functional vascularization using eMSOT imaging. We focused our investigation on breast cancer and, thus, employed the KPL-4 (ER^-^/HER2^+^) cell line as a preclinical tumor model of aggressive breast cancer. All the results presented in the prospective study are obtained from a single mouse cohort in which KPL-4 tumor-bearing mice were randomized into two treatment groups prior to the initiation of the MSOT imaging experiment and Taxotere therapy. The *in vivo* MSOT/eMSOT data acquired on the final imaging session were compared with the findings of *ex vivo* cryosectioning and IHC analysis of tumors obtained at the end of the imaging experiment. All data obtained for this project are shown in this manuscript, and no mice were excluded from analyses, even if they were outliers. Details of animal age and sex are provided below. Group numbers of mice used in *in vivo* MSOT imaging and *ex vivo* histologic experiments are reported below and in the Figure legends.

### Compounds

Clinical grade docetaxel (Taxotere®; Sanofi, Paris, France) was obtained as a stock solution of 20 mg/mL and was diluted with sterile saline (B. Braun, Germany).

### Cell lines

The human breast adenocarcinoma cell line KPL-4 was kindly provided by J. Kurebayashi (Kawasaki Medical School, Kurashiki, Japan). KPL-4 cells were cultured in Dulbecco's modified Eagle's medium (Sigma), supplemented with 10% FBS (Gibco) and 1% pen strep (Sigma). Cells were cultured at 37°C and 5% CO_2_ and routinely passaged twice weekly until they reached the final cell concentration for injection. The cells were authenticated by the American type culture collection (ATCC) and analysed by several tests for post-freeze viability, morphology, post-freeze cell growth, interspecies determination and PCR testing of cell culture media for bacterial and fungal contamination, according to the guidelines issued by the national accreditation body for the Federal Republic of Germany (Deutsche Akkreditierungsstelle (DAkkS)). Additional mycoplasma contamination tests were performed using a commercial mycoplasma detection kit (MycoAlertTM, Lonza, Basel, Switzerland). Cells were used for implantation after 3-4 passages following thawing from frozen stocks.

### Animals

All procedures involving animal experiments were approved by the Government of Upper Bavaria. All animal experiments were performed in 6-8-week-old, adult female, hairless SHrN® (NOD.Cg-PrkdcscidHrhr/NCrHsd) NOD.SCID mice (Envigo, Huntingdon, United Kingdom). Animals were housed in experimental animal rooms under specified pathogen-free (SPF) conditions with a 12 h light/dark cycle. The animal rooms are fully air-conditioned, with target values set to 20-24 °C temperature and 45-65% air humidity in accordance with Annex A of the European Convention 2007/526 EG. The maximum stocking densities correspond to Annex III of Directive 2010/63/ EU. If the animals are intolerant, the stocking density is reduced. Cages are equipped with laboratory animal bedding (wood fiber/chips, e.g. Lignocel Select Fine, Rettenmeier). To improve the housing conditions (enrichment), the cages are filled with autoclaved nesting material (mainly nestlets, cardboard houses, pulp). The cages are changed weekly on average, more often in the case of heavy soiling, and less frequently in the case of low soiling or fresh litters in order to disturb the animals as little as possible. The animals received sterile filtered water and a standard diet for rodents (e.g. Altromin 1314) *ad libitum*. Animals were allowed to acclimate for 1 week prior to experiments. General animal health conditions were monitored daily, signs of distress and body weight were monitored twice weekly and tumor volume 1-2 times per week. Animals were euthanized when tumors reached 1.5 cm in diameter or developed ulceration, in accordance with the termination criteria set by our institute's animal protocol.

### Experimental Model

**Figure 1:**
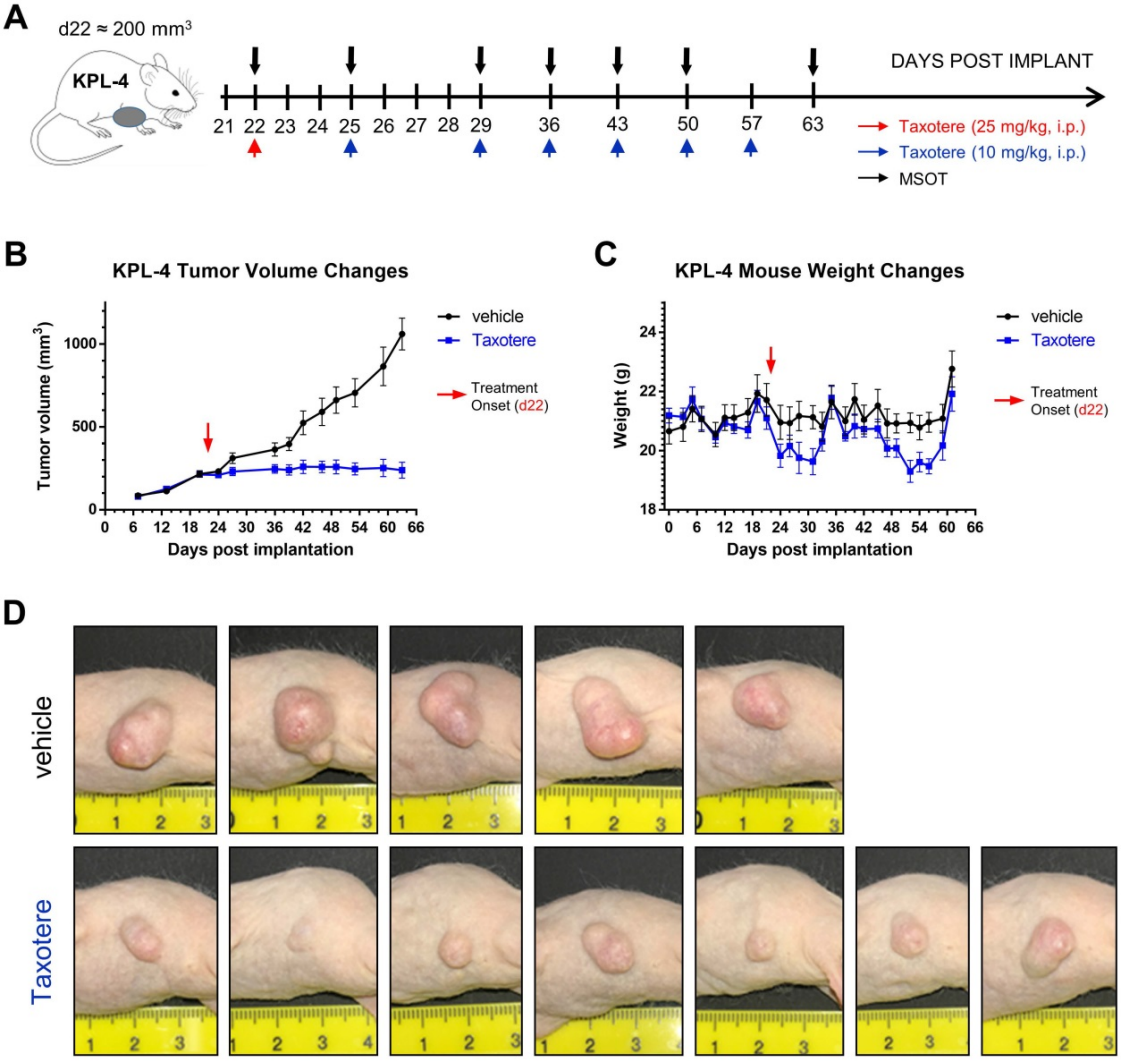
** Taxotere inhibits the growth of KPL-4 breast tumor xenografts. (A)** Schematic illustrating MSOT imaging and treatment scheduling. KPL-4 tumor bearing mice (n = 7 per treatment group) were injected on d22 post implantation with vehicle (saline) or a high priming dose of Taxotere (25 mg/kg, i.p.) (red arrow), after which vehicle or Taxotere (10 mg/kg, i.p.) was administered at the indicated time points (blue arrows). **(B)** Tumor growth curves for mice treated with saline (black line) or Taxotere (blue line).** (C)** Weight changes for saline (black line) and Taxotere (blue line) treated KPL-4 tumor-bearing mice. **(D)** Photographs of KPL-4 tumors of sacrificed mice obtained at the end of the imaging experiment (day 63 post implantation). Values in graphical plots are reported as mean ± SD (n = 7 mice per treatment group). *2 vehicle-treated mice were sacrificed at earlier time points (d36 and d42 post implantation) due to development of tumor ulceration (see Methods).

All experimental procedures were performed under aseptic conditions and in accordance with protocols approved by the Institutional Animal Care and Use Committee. For tumor cell implantation, cells were harvested using 0.05% trypsin, washed and centrifuged in culture medium. KPL-4 cells were re-suspended in phosphate-buffered saline (PBS) at a concentration of 3 x 10^6^ cells/50 µl and then mixed with 50 µl Matrigel (Corning, NY) to produce a 100 µl 1:1 mixture of PBS and Matrigel. Cell suspensions containing 3 x 10^6^ KPL-4 cells were inoculated orthotopically into the right third thoracic mammary fat pad of mice (n = 7 per KPL-4 treatment group) with a tumor take of 100%. All tumor inoculation procedures were completed with the animal anesthetized under isoflurane. KPL-4 tumors were allowed to grow for 22 days from the time of implantation when the mean volume reached ≈ 200 mm^3^, after which animals were randomized into two groups; 1) vehicle group, receiving warm saline injections (i.p.) on days 22, 25, 29, 36, 43, 50 and 57 post implantation, 2) Taxotere group, treated with an initial priming dose of Taxotere (25 mg/kg, i.p) on d22 post implant, followed by subsequent doses (10 mg/kg, i.p) on days 25, 29, 36, 43, 50 and 57 post implantation (Figure 1A). Treatment was initiated immediately following the first MSOT imaging session. All mice survived until the end time point of the imaging experiment (d63 p.i.), except for 2 vehicle-treated KPL-4 tumor-bearing mice that had to be sacrificed at earlier time points (d36 and d42 p.i.) due to tumor ulceration. Tumor growth was measured in two dimensions using vernier calipers, and tumor volume was calculated using the ellipsoid formula; V = D x d2 x 0.5, where D is the longest diameter and d is the perpendicular short diameter. Growth curves were obtained by plotting mean tumor volume against time. Relative tumor volume (RTV) of individual tumors was calculated using the following formula: RTV = Vx/V_1_ where Vx is the volume in mm^3^ at the end and V_1_ at the start of treatment. The percentage tumor growth inhibition (TGI) was calculated as the mean (RTV of vehicle-treated mice - RTV of Taxotere-treated mice/mean RTV of vehicle-treated mice) x 100.

### MSOT imaging of KPL-4 tumors

The MSOT system and animal preparation procedures were described in detail previously 32, 36. Briefly, volumetric optoacoustic imaging was performed using a commercial, real-time whole-body mouse imaging system, MSOT inVision 256-TF (iThera-Medical GmbH, Munich, Germany). All imaging procedures were performed under anesthesia using 2% isoflurane (Zoetis GmbH, Berlin, Germany) delivered in combination with oxygen. Mice were lying in the prone position during scanning. MSOT imaging of KPL-4 tumors was initiated on d22 post implant. KPL-4 tumor-bearing mice were imaged at 7 different time points, on days 22, 25, 29, 36, 43, 50, and 63 post implantation (Figure 1A). MSOT scanning was performed at 21 wavelengths from 700 to 900 nm with a step size of 10 nm, and 6-35 consecutive slices (depending on tumor size) were acquired for each tumor with a step size of 0.3 mm. MSOT image acquisition was performed by acquiring 10 average frames per wavelength and, therefore, the total duration for acquiring a single multispectral optoacoustic frame was ≈ 29 sec. Image reconstruction was performed using a model-based inversion algorithm with a non-negativity constraint imposed during inversion and with Tikhonov regularization, as described before 41, 42.

### eMSOT image processing and data analysis

All optoacoustic image generation and processing was performed using a custom graphical user interface developed in Matlab (Mathworks, MA, USA), that employs the eMSOT algorithm as described previously 36. Briefly, whole tumor sO_2_ and THb content was calculated volumetrically using the entire set of optoacoustic slices (image stack) obtained from each scan. To calculate the averaged tumor sO_2_ values, a region of interest (ROI) encompassing the entire tumor area was manually drawn for each eMSOT image and successively adjusted between consecutive frames. Mean total hemoglobin concentration (THb) was calculated using the 800 nm optoacoustic images which correspond to the isosbestic point of oxy- and deoxyhemoglobin. sO_2_ and THb mean intensity values derived from each optoacoustic frame were recorded in excel sheets and averaged and the averaged sO_2_ and THb values from all mice of each experimental group (n = 7) were calculated and plotted as a function of time. All color maps representing % sO_2_ signal were displayed using a color lookup table superimposed on corresponding anatomical (THb) images. In the resulting optoacoustic eMSOT maps, black pixels correspond to areas where the hemoglobin signal was too low to obtain a reliable spectral fitting, and are indicative of necrotic or poorly vascularized/perfused viable tumor regions. Tumor regions containing such black pixels were excluded from all sO_2_/THb calculations. All data analysis, including image ROI drawing, was performed blinded until the final statistics were obtained, and no outliers were excluded from analysis.

### Oxygen enhanced eMSOT (OE-eMSOT) imaging

OE-eMSOT imaging was performed on the final MSOT imaging session (d63 post implantation) immediately after static eMSOT imaging without interrupting anesthesia between static and OE-eMSOT imaging experiments. Mice were allowed to equilibrate in medical air (21% oxygen) mixed with isoflurane for 10 min before initiating MSOT image acquisition. Next, MSOT imaging was performed by acquiring multispectral optoacoustic frames of a single central tumor plane (the cross-sectional tumor plane with the largest diameter) under medical air (21% oxygen) and 100% oxygen conditions. A total of 9 optoacoustic frames were acquired while breathing medical air over a time span of 4.5 min, then the breathing gas was immediately switched manually from 21% oxygen to 100% oxygen using separate flow meters and 11 more frames were acquired for an additional 5.5 min. For each optoacoustic frame 21 wavelengths were acquired (700-900 nm) with 10 averages per frame and the average duration for acquiring a single MSOT frame was approximately 29 sec. Following optoacoustic image reconstruction and processing using the eMSOT algorithm (see eMSOT image processing and data analysis above), measurements were extracted in the form of oxygen saturation (sO_2_). sO_2_ was quantified in three tumor regions (whole tumor, rim, core) by subregion segmentation analysis of eMSOT-sO_2_ images in ImageJ (imageJ.nih.gov) as described in 32. Briefly, segmentation was performed by manually tracing ROIs encompassing each tumor subregion. The “rim” was defined as the perimeter area of 1 mm depth into the tumor for tumors < 1 cm in diameter and of 2 mm depth in tumors > 1 cm in diameter. The “core” was defined as the remaining tumor area after the rim was excluded. Mean whole tumor sO_2_ values during 21% and 100% breathing for each treatment group were calculated from the non-linear fits of the data. The change in sO_2_ (ΔsO_2_) was calculated by subtracting the mean whole tumor sO_2_ (derived from the sigmoidal curves) while breathing medical air from the mean tumor sO_2_ while breathing 100% oxygen.

### Histologic tumor analysis

Snap-freezing of excised tumors and tumor cryosectioning were described previously 32.

### Immunohistochemical staining of tumor cryosections

The cryosections were fixed in in ice-cold acetone for 10 min followed by 3 x 5 min washes in PBS. Then, the tumor sections were blocked with 10% goat serum (in PBS) for 1 h and incubated for 30 min with Crystal A MausBlock reagent (Innovative Diagnostik-Systeme, Hamburg, Germany) at room temperature, washed 3 x 2 min with PBS. Following, the sections were incubated with Crystal B MausBlock reagent (Innovative Diagnostik-Systeme, Hamburg, Germany) for 5 min at room temperature and washed again 3 x 2 min in PBS. Afterwards, the slides were incubated overnight at 4 °C with mouse MAB1 (1:50, Hydroxyprobe Inc., MA, USA), rat CD31 (1:20, Dianova Research, Hamburg, Germany) and rabbit Carbonic Anhydrase 9 (1:100, Abcam, UK) antibodies diluted in PBS containing 0.1% BSA plus 1% goat at serum plus 0.1% Tween-20. Subsequently, the slides were washed 3 x 5 min with PBS and then were incubated with AF488 anti-mouse, AF594 anti-rabbit and AF680 anti-rat antibodies (1:500, ThermoFisher Scientific, MA, US) diluted in 1% goat serum for 1 h, at room temperature. Then the slides were washed 3 x 5 min in PBS and mounted with coverslips using Prolong Diamond mounting agent (ThermoFisher Scientific, MA, US).

### Fluorescence microscopy imaging

Representative slides were imaged using a Zeiss Axio Imager M2 microscope fitted with an AxioCam 105 color camera and pictures were then processed using a motorized stitching Zen Imaging Software (Carl Zeiss Microscopes GmbH, Jena, Germany). Fluorescent images were captured under identical conditions (exposure time, scaling) and thresholded to exclude background signal from secondary antibody alone.

### Spatial co-registration of optoacoustic and histopathological data

Registration of THb-CD31 and eMSOT-sO_2_-pimonidazole image pairs was performed using the Big Warp plugin in ImageJ (imageJ.nih.gov) as described before 32.

### Calculation of microvessel density, hypoxic and necrotic fractions

Microvessel density (MVD) was assessed by fluorescent microscopic analysis of CD31-stained tumor sections as described previously 32. Briefly, areas of highest neovascularization (neovascular "hot spots") were identified under low (40x) magnification and then individual microvessels were counted on a 100x field equal to 1 mm^2^ in area using a calibrated grid. The mean of 4 single 100x fields was computed for each CD31-stained whole tumor cross-section and results are expressed as the average number of microvessels from 7-8 central sections per tumor.

Tumor hypoxia was determined by area fraction analysis of binary thresholded pimonidazole-stained whole tumor cross-sectional micrographs. Thresholding was performed using the Automatic Threshold plugin in ImageJ (imageJ.nih.gov), which employs the Otsu 43 thresholding method to assign global (histogram-derived) thresholding values to fully-stitched, grey scale pimonidazole fluorescence micrographs. Hypoxic fraction (HF) was calculated as the percentage of the positive pimonidazole fluorescent area divided by the total vital tumor tissue area, with the necrotic areas excluded. The methodological approach followed for the calculation of hypoxic area fraction is summarized in Figure S1A-F. The HF for each tumor was calculated from 7-8 pimonidazole-stained central whole tumor cross-sections (with the tumor rim area excluded). All HF quantification data presented in Figure 4G and Figure 4J were calculated by Automatic Thresholding analysis, as described above.

Necrotic areas were identified in DAPI nuclear stains from 7-8 central sections per tumor and defined as tumor regions with complete absence of DAPI staining including regions with condensed nuclei debris. Necrotic areas were segmented as regions of interest by hand in ImageJ (imageJ.nih.gov). Tumor necrotic fraction (NF) was defined as the surface area of necrotic tissue divided by the total tumor area (excluding the vascular rim). The MVD, HF and NF values reported in corresponding bar graphs are the average of all tumors analyzed (n = 5 for vehicle and n = 7 for Taxotere treatment group, respectively).

### Statistical analysis

Statistical analyses and graphical representation of data were performed using Graphpad Prism 6 (GraphPad Software, San Diego, CA) (Microsoft, WA, USA). All results in this work are reported as mean ± SD. Statistical differences in sO_2_ between treatment groups were assessed by unpaired two-tailed t-test. Non-linear fitting of the averaged kinetic sO_2_ curves was performed in Graphpad Prism 6. Pearson correlation analysis was performed on a per-tumor-basis using pooled HF and volumetric whole tumor eMSOT- sO_2_ measurements from KPL-4 vehicle (n = 5) and Taxotere-treated (n = 7) mice.

## Results

### Taxotere suppresses KPL-4 tumor growth

Taxotere treatment and MSOT imaging scheduling are summarized in Figure 1A. KPL-4 tumor-bearing mice were treated with vehicle (saline) or Taxotere on the indicated days (red and blue arrows, Figure 1A). MSOT imaging was performed immediately prior to Taxotere administration and over the course of treatment at the indicated time points (black arrows, Figure 1A). Taxotere induced an average tumor growth inhibition (TGI) of 76.2% (Figure 1B). While all 7 mice responded to the therapy, there was some variability in the size of tumors by the end of treatment, as can be seen in the individual growth curves (Figure S2A-B). Average mouse weight loss was observed in two separate instances over the course of Taxotere treatment (Figure 1C), which was however mitigated with wet chow and was within the allowed limits (< 20% weight loss) set by our institution's animal protocol conforming to local regulatory authorities. The growth inhibitory effects of Taxotere are also evident in the tumor photographs obtained at the end of treatment (Figure 1D).

### Longitudinal static eMSOT imaging shows a reduction in tumor oxygenation as a response to Taxotere treatment

**Figure 2:**
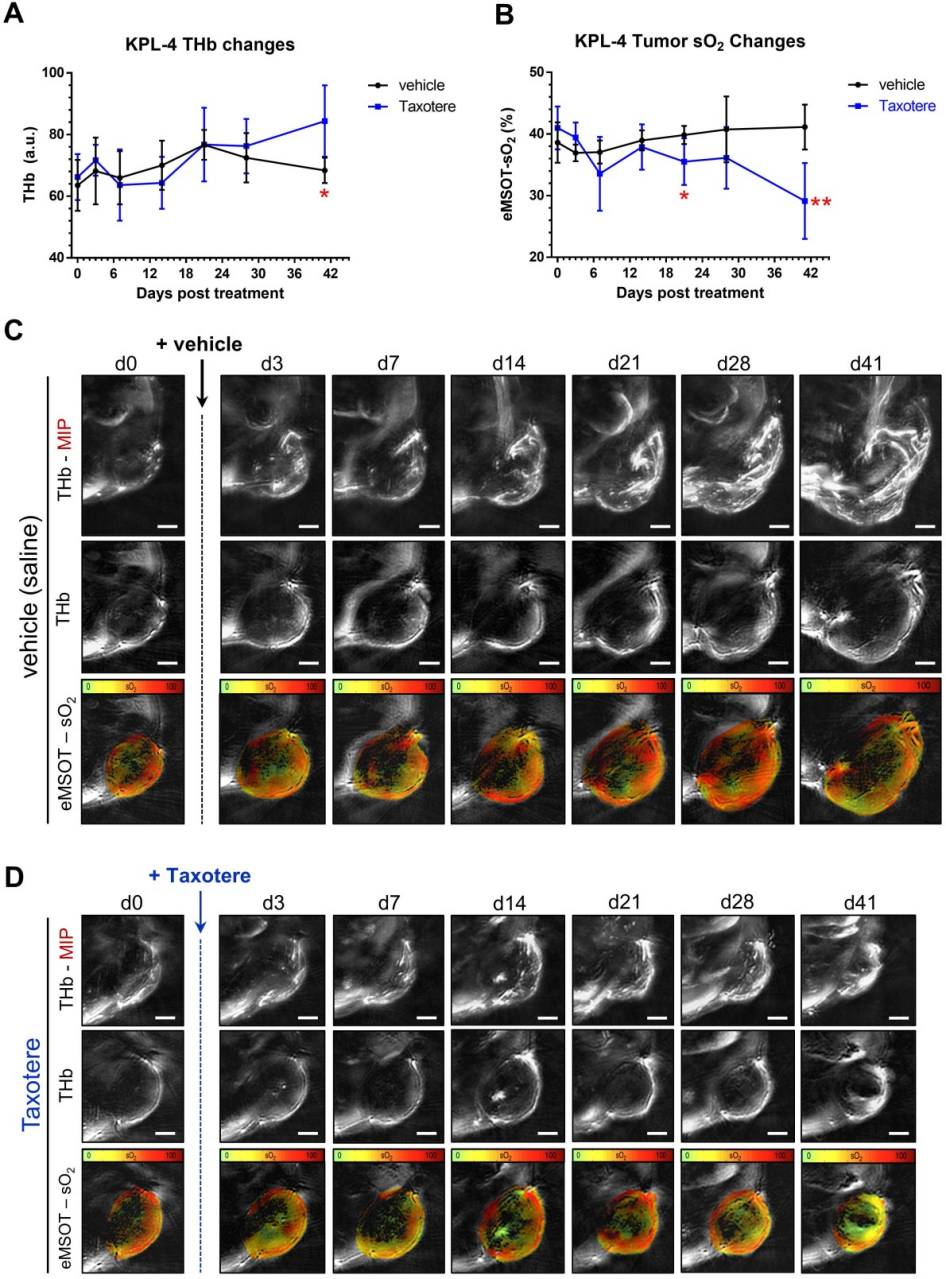
** Spatiotemporal changes in oxygenation and blood volume of KPL-4 tumors along the course of Taxotere chemotherapy assessed by MSOT/eMSOT. (A)** Mean whole tumor total hemoglobin concentration (THb) and **(B)** eMSOT oxygen saturation (sO_2_) values obtained from KPL-4 tumors of mice treated with either vehicle (saline, black lines) or Taxotere (blue lines) for days 0-41 post treatment. During the course of Taxotere therapy the average tumor sO_2_ gradually decreased, while an increase in THb was observed on the last day of the imaging experiment (d41). **(C-D)** Longitudinal changes in blood volume and oxygenation of a single representative KPL-4 tumor from vehicle **(C)** and Taxotere **(D)** treatment groups visualized by serial MSOT imaging at 7 consecutive time points. Middle panels: central optoacoustic cross-sections showing THb (800 nm, isosbestic point) distribution. Upper panels: THb maximum intensity projections (THb-MIP). Lower panels: pseudo-colorized eMSOT maps of tumor oxygen saturation overlaid on corresponding anatomical images. Color scale bars at the bottom of eMSOT images represent sO_2_ levels ranging from 0% (green) to 100% (red). Data are displayed as mean ± SD (n = 7 mice per treatment group). *p < 0.05, **p < 0.01. Statistical significance between the two treatment groups was assessed by an unpaired two-tailed t-test. Scale bar; 2 mm.

We then examined the effects of Taxotere monotherapy on tumor oxygenation and functional vascularization by performing static MSOT/eMSOT imaging longitudinally over a period of 41 days (Figure 2). Quantification of the total hemoglobin concentration (THb) revealed a rising trend in the blood volume of vehicle-treated tumors (black lines, Figure 2A), which however started declining from d21 post treatment onwards, eventually reaching pre-treatment levels by the final time point on d41. On the other hand, in Taxotere-treated animals the THb was generally lower but not significantly different from vehicle for the biggest part of the treatment duration, but became significantly higher than vehicle at the final time point (Figure 2A).

Next, we performed quantification of mean whole tumor sO_2_ in Taxotere and vehicle treated groups using MSOT images processed with the eMSOT algorithm, as shown in Figure 2B. This analysis showed that the mean whole tumor sO_2_ was relatively stable in vehicle-treated animals over the whole treatment course, while a drop in tumor sO_2_ became noticeable in the Taxotere group shortly after initiation of treatment (d7, Figure 2B). Tumor oxygenation in the Taxotere group remained lower compared to vehicle for the entire treatment duration and reached its lowest value on the last day of the experiment (d41, Figure 2B). Inter-tumoral variation in sO_2_ (Figure S3A-B) and THb (Figure S3C-D) kinetics was relatively low in both vehicle and Taxotere treated groups. Confirming these findings, the longitudinal effects of vehicle and Taxotere treatment on tumor blood volume and oxygenation can also be seen in the MSOT/eMSOT images of representative vehicle and Taxotere-treated tumors (Figure 2C-D). An additional example of an MSOT/eMSOT imaging time course is provided in Figure S4.

### Dynamic OE-eMSOT imaging reveals diminished tumor vascular response and static blood lakes in Taxotere-treated tumors

**Figure 3:**
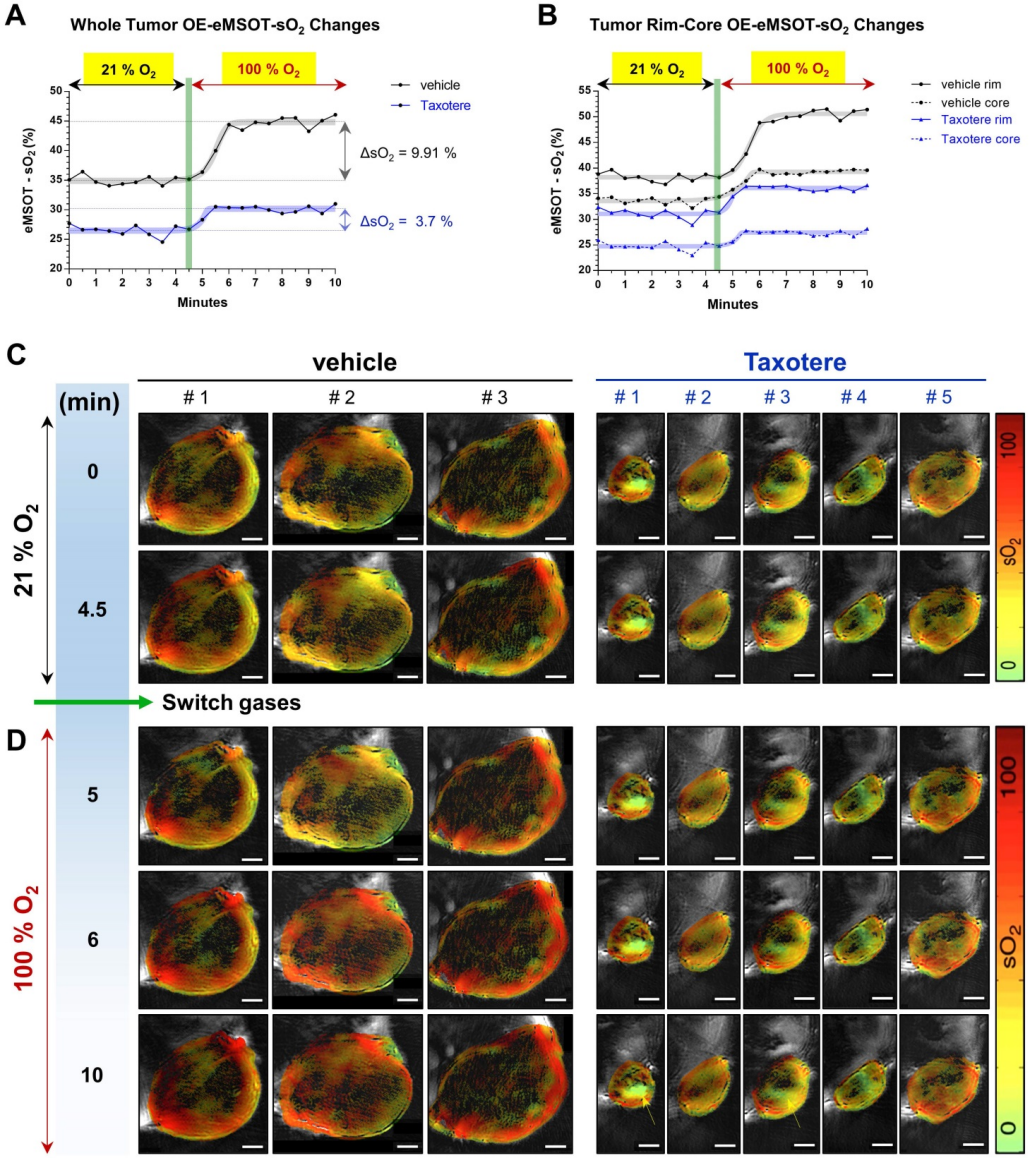
** Oxygen-enhanced eMSOT (OE-eMSOT) reveals altered oxygenation response in Taxotere-treated KPL-4 tumors subjected to an oxygen challenge. (A)** Average kinetic curves showing mean whole tumor sO_2_ values at each time point of the time course in vehicle (saline, black line) and Taxotere (blue line) treated mice subjected to an oxygen challenge (n = 5 tumors per group). The thick grey and blue sigmoidal curves show nonlinear fit of data. Taxotere-treated KPL-4 tumors exhibit little enhancement in whole tumor sO_2_ during the oxygen challenge, indicating diminished vascular function. **(B)** Average kinetic curves showing mean tumor rim (saline; black line, Taxotere; blue line) and core (saline; black dotted line, Taxotere; blue dotted line) sO_2_ values at each time point of the time course. The thick grey and blue sigmoidal curves show nonlinear fits of data. **(C-D)** eMSOT images of a single central optoacoustic tumor cross-sections from representative vehicle (3 left panels) and Taxotere (5 right panels) treated mice acquired during medical air (21% O_2_) **(C)** and pure oxygen (100% O_2_) **(D)** breathing conditions. Bottom panels: The presence of hemorrhagic blood pools in Taxotere-treated tumors #1 and #3 is indicated by yellow arrows. Color scale bars on the right of eMSOT images represent sO_2_ levels ranging from 0% (green) to 100% (red). The switch from 21% to 100% O_2_ is denoted by a vertical green line. Scale bars; 2 mm.

In order to gain a better insight into the vascular changes induced by Taxotere chemotherapy, we performed oxygen-enhanced eMSOT (OE-eMSOT) imaging on the final day (d63 post implantation) of the experiment (Figure 3). OE-eMSOT imaging was conducted by acquiring a total of 21 multispectral optoacoustic frames of central tumor planes before and during an oxygen challenge, as described in detail in Material and Methods. The temporal evolution of sO_2_ in KPL-4 vehicle and Taxotere-treated tumors was determined by eMSOT image quantification, as shown in Figure 3A. The mean whole tumor sO_2_ was significantly lower in Taxotere-treated mice prior to the challenge compared to vehicle (sO_2_ vehicle vs sO_2_ Taxotere = 34.92% vs 26.48%, p < 0.0001). Switching from 21% to 100% oxygen caused an sO_2_ increase (ΔsO_2_) of 9.91% in vehicle-treated tumors which peaked after approximately 1.5 min, after which the sO_2_ levels reached a plateau (black curve, Figure 3A). Moreover, the sO_2_ kinetics of individual vehicle-treated tumors fluctuated considerably over the time span of the experiment and there was notable inter-tumoral heterogeneity in response to the challenge (Figure S5A). By contrast, the amplitude of the ΔsO_2_ response in tumors of the Taxotere group was considerably lower on average (ΔsO_2_ = 3.7%, Figure 3A) with relatively low inter-tumoral variability, as shown in the individual whole tumor sO_2_ kinetic curves (Figure S5B).

The increase in oxygen saturation was more prominent at the periphery of vehicle and Taxotere-treated tumors, while core tumor regions were less responsive to the challenge (Figure 3B). Specifically, vehicle-treated tumors displayed a substantial enhancement in sO_2_ at their rims (ΔsO_2_ = 12.37%), while hypoxic areas at their core showed a less pronounced enhancement (ΔsO_2_ = 5.7%) in response to the challenge (Figure 3B). On the other hand, tumors of the Taxotere group exhibited a significantly reduced response compared to vehicle both at the rim (ΔsO_2_ = 5.05%) and core (ΔsO_2_ = 2.68%) subregions after the change to 100% oxygen (Figure 3B), indicating functional disruption of the vasculature due to the treatment. The inter-tumoral variability at the rim and core sO_2_ of tumors treated with saline or Taxotere is shown in the respective individual kinetic curves (Figure S5C-F). These sO_2_ changes in vehicle and Taxotere-treated tumors in response to the oxygen challenge can also be clearly appreciated in the representative eMSOT images (Figure 3C-D) and time-lapse video (Movie S1). Furthermore, while no blood lakes were detected in any of the tumors from vehicle-treated mice (Figure S6A), the presence of stagnant blood pools that show no obvious response to the challenge was observed in a subset of KPL-4 tumors (tumors #1 and #3) from Taxotere-treated mice (Figure 3C-D and Figure S6B, yellow arrows).

### Immunohistochemical analysis validates *in vivo* MSOT/eMSOT data and reveals the presence of blood lakes in Taxotere-treated tumors

**Figure 4:**
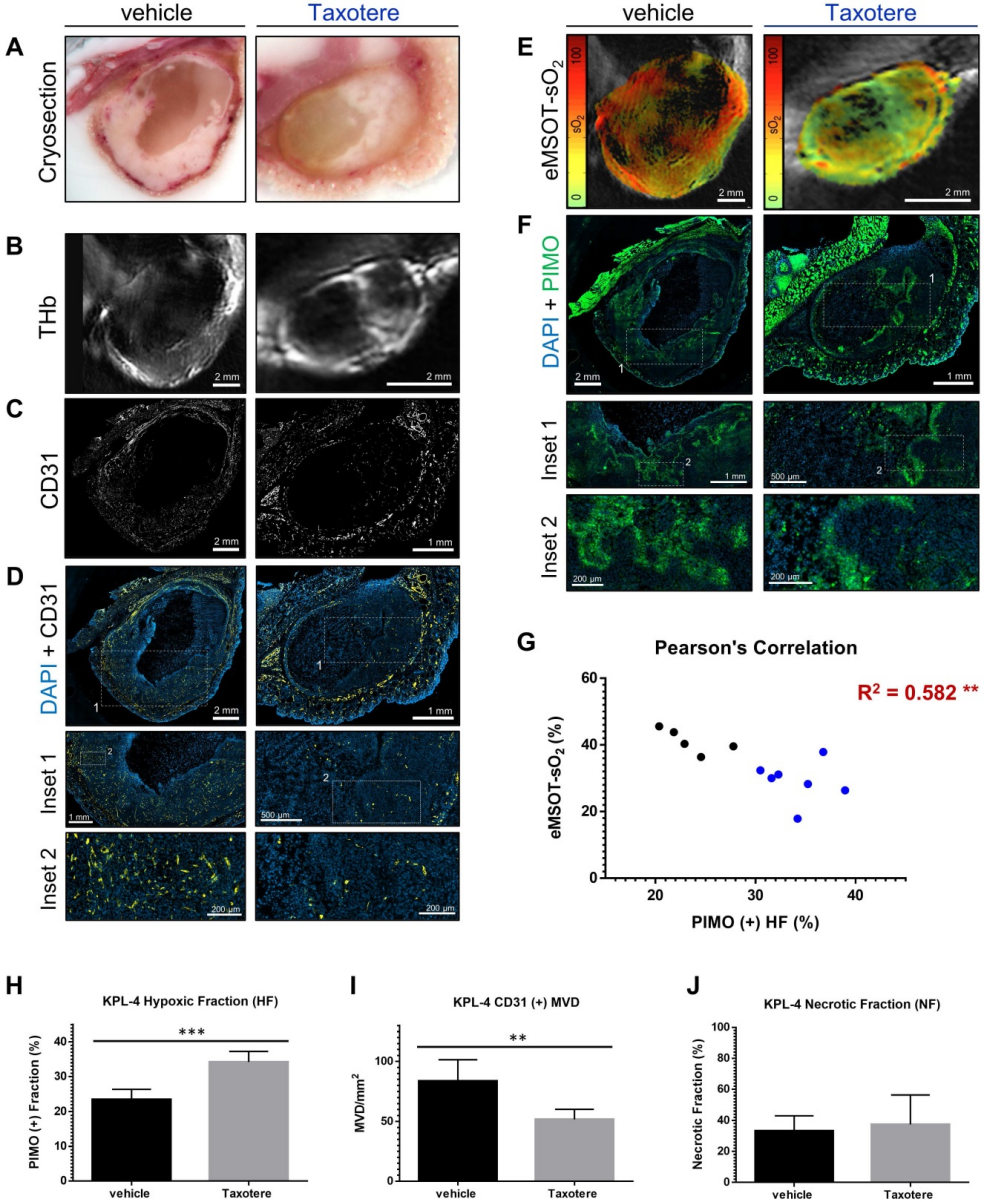
** Histopathologic assessment of tumor hypoxia and vascularity confirms *in vivo* optoacoustic data. (A-F)** Micrographs of central histologic whole tumor cross sections and corresponding optoacoustic slices from representative vehicle (left panels) and Taxotere-treated (right panels) tumors; **(A)** Cryosection block color photography. **(B)** THb distribution (800 nm, isosbestic point). **(C)** CD31+ microvessel distribution. **(D)** DAPI nuclear staining (blue) merged with CD31 (yellow) immunofluorescence signal. **(E)** eMSOT mapping of tumor oxygen saturation (sO_2_) overlaid on corresponding anatomical images. Color scale bars on the left of eMSOT images indicate sO_2_ levels ranging from 0% (green) to 100% (red). **(F)** DAPI (blue) merged with pimonidazole (green) immunofluorescence. Insets 1 and 2 in (D) and (F) represent successively magnified regions enclosed in the corresponding white-dotted rectangles. **(G)** Pearson's correlation analysis of optoacoustic and histopathologic data sets showing inverse correlation (R^2^ = 0.582) between pimonidazole-positive hypoxic fraction (HF) and whole tumor eMSOT-sO_2_ estimates. Correlations were assessed on a per-tumor basis from pooled HF/eMSOT-sO_2_ measurements from vehicle (black dots) and Taxotere-treated (blue dots) mice (see Materials and Methods). **(H)** Tumor hypoxic fraction (HF).** (I)** CD31+ microvessel density (MVD). **(J)** Tumor necrotic fraction (NF). Data are displayed as mean ± SD (n = 5 mice for vehicle and n = 7 for Taxotere-treated groups respectively). **p < 0.01, ***p < 0.001. Statistical significance between the two treatment groups was assessed by an unpaired two-tailed t-test. Scale bar; 2 mm.

To validate the *in vivo* optoacoustic findings and evaluate the effects of Taxotere treatment on tumor vasculature and hypoxia to a greater detail, we conducted immunohistochemical analysis using the endothelial marker CD31 and two hypoxia markers (pimonidazole, CA9). Figure 4 shows images of co-registered histologic and optoacoustic cross-sections from a representative saline and a Taxotere-treated tumor harvested at the end of the imaging experiment. The cryosection images (Figure 4A) show overall tissue morphology and the presence of large necrotic areas devoid of hemorrhage at the cores of vehicle and Taxotere-treated tumors. Moreover, the distribution of THb in tumors of either group (Figure 4B) is closely matched to the vascular morphology revealed by CD31 staining (Figure 4C). Further visual inspection reveals that the microvessel density (MVD) in the viable tumor tissue is substantially reduced in Taxotere-treated tumors compared with vehicle (Figure 4D).

As a next step, we compared the spatial distribution of eMSOT-derived hemoglobin oxygen saturation and tissue hypoxia determined by pimonidazole staining (Figure 4E-F). The hypoxemic patterns resolved by eMSOT (Figure 4E) appear close to the corresponding hypoxia patterns revealed by pimonidazole staining (Figure 4F). Detailed examination of pimonidazole stains shows that these hypoxic areas are localized perinecrotically in tumors in both treatment groups (Figure 4F, insets 1-2). In addition, an inverse correlation was found between pooled eMSOT-derived estimates of whole tumor sO_2_ and hypoxic fraction on a per-tumor basis (Figure 4G) and the hypoxic fraction was significantly higher in the Taxotere group compared to vehicle (Figure 4H). Quantification of MVD showed a significantly lower microvessel density in tumors treated with Taxotere compared with vehicle (Figure 4I). Tumor necrosis was higher in Taxotere-treated animals, although the difference compared to vehicle was not statistically significant (Figure 4J).

**Figure 5:**
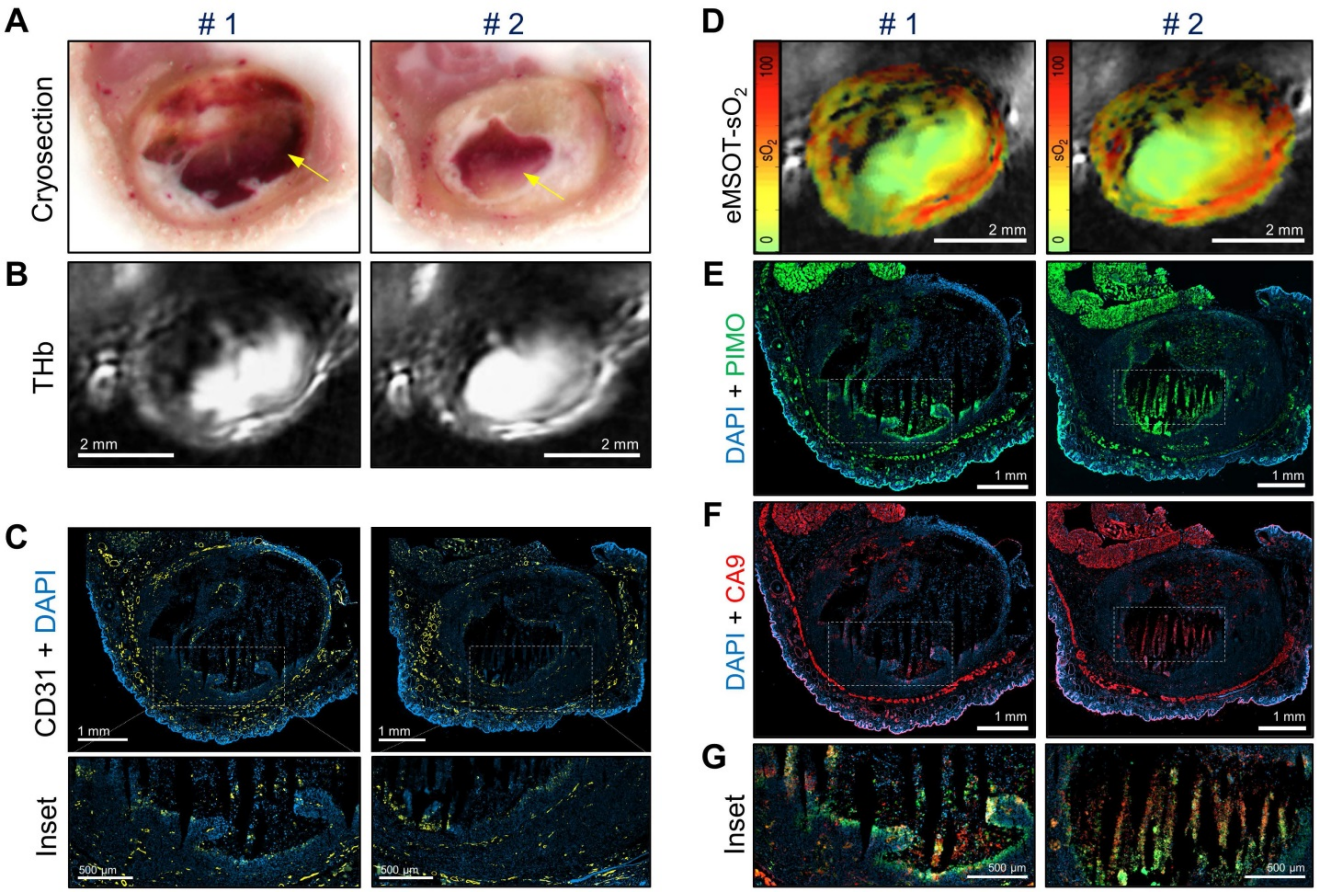
** Taxotere induces the formation of blood lakes in KPL-4 tumors.** Co-registered histologic and optoacoustic image pairs of two distinct transversal planes obtained from a single KPL-4 tumor at the end of Taxotere treatment. The two cross-sectional tumor planes (denoted by #1 and #2) are spaced by 1 mm. **(A)** Cryosection block color photography. The presence of a blood lake in tumor cryosections is indicated by a yellow arrow. **(B)** MSOT images showing THb distribution (800 nm, isosbestic point). **(C)** CD31+ immunofluorescence (yellow) merged with DAPI fluorescence (blue). Insets are magnified views of the corresponding regions enclosed in the white-dotted rectangles. **(D)** eMSOT mapping of tumor oxygen saturation (sO_2_) shows that the blood lakes in #1 and #2 are filled with de-oxygenated blood. Color scale bars on the left of eMSOT images indicate sO_2_ levels ranging from 0% (green) to 100% (red). **(E)** pimonidazole immunofluorescence (green) overlaid with DAPI (blue). **(F)** CA9 immunofluorescence (red) overlaid with DAPI (blue). **(G)** Magnified insets of the corresponding white-dotted rectangles in (E) and (F) showing merged DAPI, pimonidazole and CA9 immunofluorescence.

Figure 5 shows co-registered optoacoustic and histologic images of a single KPL-4 tumor harvested at the end of Taxotere treatment. The presence of large hemorrhagic areas (blood lakes) is evident in both cross-sectional planes (denoted by #1 and #2) of the tumor (Figure 5). Such hemorrhagic tumor regions have a characteristic dark red appearance in tumor cryosections (yellow arrows, Figure 5A) and also produce a homogeneous, bright optoacoustic signal in THb images that can be easily distinguished from vascular structures (Figure 5B). Furthermore, the overall geometry of these blood pools appears similar between cryosection photographs and THb images in both transversal tumor planes examined, demonstrating the sectioning capabilities of MSOT. Detailed immunohistochemical analysis shows that these blood lakes consist of large necrotic regions with condensed nuclei debris (revealed by DAPI) and are devoid of CD31-positive blood vessels (Figure 5C). In addition, eMSOT imaging demonstrates that the vascular lakes are filled with de-oxygenated blood (Figure 5D). Immunohistochemical staining showed high accumulation of pimonidazole adducts in tumor cells localized peripherally to the blood lakes as well as in some apoptotic/necrotic cells present inside the blood pools (Figure 5E). Anti-CA9 staining demonstrated similar hypoxia patterns with pimonidazole (Figure 5F), while high immunofluorescence signal co-localization was found between the two hypoxia markers (Figure 5G and Figure S7).

## Discussion

While the adverse effects of the TME on taxane therapeutic efficacy and resistance are becoming increasingly understood, it is not clear how taxanes reshape the TME and even less is known about the functional consequences of taxane treatment on tumor oxygenation and hemodynamics. Moreover, to our knowledge, no previous study has examined the tumor oxygenation responses to Taxotere therapy in a longitudinal fashion. In this study, we sought to determine the dynamic effects of Taxotere chemotherapy on KPL-4 breast tumor oxygenation and vascular functionality using static longitudinal MSOT/eMSOT imaging similar to a previous study by our group that examined the longitudinal effects of bevacizumab on breast tumor hemodynamics 32. In the current work, we additionally employed for the first time a variation of the oxygen-enhanced MSOT imaging method 27, 39, 40 that we named “oxygen-enhanced eMSOT (OE-eMSOT) imaging” to gain insights into tumor vascular function in response to Taxotere therapy. This novel dynamic optoacoustic imaging technique applies the eigenspectra unmixing approach 36, 37 to temporally resolved MSOT measurements, yielding substantially improved estimates of blood oxygenation changes in response to an oxygen challenge compared with linear unmixing methods. Furthermore, to our knowledge, this is the first time an oxygen-enhanced optoacoustic method is implemented in the context of a chemotherapeutic study.

Our combined optoacoustic and histologic results suggest that Taxotere chemotherapy induces both anti-angiogenic and vascular disruption effects in KPL-4 tumors. In particular, OE-eMSOT showed a diminished response to the oxygen challenge in Taxotere-treated tumors at both the rim and core subregions, suggesting deficient vascular function in response to the treatment. Likewise, quantitative histopathologic analysis showed a significantly reduced microvessel density in tumors subjected to Taxotere treatment. Collectively, these findings are consistent with previous studies in endothelial cell cultures and animal tumor models 44-50 demonstrating the potent anti-angiogenic activity of taxanes.

While the anti-vascular and anti-angiogenic properties of taxanes are well established, the functional consequences of taxane chemotherapy on tumor oxygenation are largely unknown. Here, we report for the first time that Taxotere causes a significant drop in oxygen saturation after prolonged treatment in a mouse breast tumor xenograft model (KPL-4). In particular, longitudinal static eMSOT imaging showed a progressive reduction in tumor sO_2_ in response to Taxotere administration that became significantly more pronounced compared to vehicle towards the end of therapy. This finding was further corroborated by dynamic OE-eMSOT measurements showing significantly reduced oxygenation responses in tumors subjected to Taxotere treatment.

Although, to our knowledge, no study has previously investigated the effects of Taxotere monotherapy on tumor oxygenation, our findings are in disagreement with several earlier studies reporting elevated tumor oxygenation in mouse mammary carcinoma xenografts shortly (24-72 h) after paclitaxel treatment 51, 52 and in breast cancer patients at the end of neoadjuvant paclitaxel therapy 53 based on pO_2_ polarography. While we did not detect a significant increase in tumor sO_2_ at any time point of the Taxotere treatment course, we do not preclude the possibility that transient (cyclic) increases in tumor oxygenation and blood volume may account for some of the findings of the abovementioned polarographic studies. On the other hand, our results are in line with previous clinical imaging studies 54-57 that reported a general increase in tumor hypoxia in breast cancer patients receiving taxane treatment as part of combined NACT neoadjuvant chemotherapy (NACT), particularly in responders. Thus, given the wide variability in experimental parameters such as type of taxane formulation tested (e.g. paclitaxel, nab-paclitaxel, docetaxel, Taxotere), dosage, scheduling, method of detection and type of tumor examined, it is difficult to draw direct comparisons with previous studies regarding the effects of taxanes on tumor oxygenation.

Differences in the non-linear attenuation of light as a function of depth and wavelength pose a challenge to the quantification of chromophore concentrations and oxygenation in bulk tissue. In particular, the application of linear unmixing techniques to multi-wavelength optoacoustic data has been shown to lead to gross quantification errors of tissue oxygenation and corresponding vascularization, especially as the depth of the observations increases 36, 37. Numerous approaches for compensation of wavelength-dependent fluence attenuation have been previously proposed, including the optical transport model, the diffuse optical tomography (DOT) enhanced method, the acoustic spectrum-based method and others (for a recent review, see 58). A major advantage of the eMSOT technique is that it corrects for the effects of light attenuation and spectral coloring in tissue by modeling several fundamental optical absorption spectra (eigenspectra) in tissue, thus solving the spectral unmixing problem in the spectral domain instead of the spatial domain 36. This results in superior performance to linear unmixing methods at increased imaging depths 36, 37, effectively accounting for the differential light attenuation and spectral coloring in tissue.

In the present study, the large control tumors are expected to have substantially different optical attenuation profiles compared to the smaller Taxotere-treated tumors by the end of the therapeutic course. Our results suggest that the significant difference in whole tumor sO_2_ between control and Taxotere-treated groups observed at the end of the therapeutic course (d41, p < 0.01, Figure 2B) is attributed to specific effects of Taxotere therapy and is not caused by size-dependent differential light attenuation. In support of this conclusion, at the pre-treatment time point (d0), when tumors of vehicle and Taxotere groups had an almost identical average size (d0, vehicle vs Taxotere: 86.4 vs 80.8 mm^3^, Figure 1B), it is expected that the two groups also exhibited similar light attenuation profiles and thus similar mean tumor sO_2_. Indeed, our results show that the mean sO_2_ was comparable between the two groups at pre-treatment (d0, vehicle vs Taxotere: 38.6 vs 41.0 %, p > 0.05, Figure 2B), validating eMSOT's ability to generate reproducible sO_2_ values in similarly sized tumors in the absence of therapy. Another finding that supports this conclusion is that by the end of therapy (d41) tumors of the Taxotere group had significantly lower mean sO_2_ compared to pre-treatment (d0 vs d41: 41.0 vs 29.1 %, p < 0.01, Figure 2B), despite their similar size (d0 vs d41: 80.8 vs 239.4 mm^3^, Figure 1B). This result implies that the difference in sO_2_ between start and end time points cannot be simply attributed to differential light attenuation and that other Taxotere-specific effects (such as the demonstrated inhibition of functional vascularization, Figure 3) account for the observed drop in tumor oxygenation. Moreover, while the present study demonstrated a significant drop in mean KPL-4 tumor sO_2_ levels by the end of Taxotere therapy, we previously showed 32 that another drug (bevacizumab) caused an increase in mean sO_2_ in the same tumor model. Thus, while both drugs caused a substantial KPL-4 tumor growth inhibition, they had diametrically opposite effects on tumor oxygenation, further verifying that the changes seen in sO_2_ are therapy-specific and not simply due to tumor size-dependent spectral coloring effects. Altogether, these findings confirm eMSOT's ability to provide unprecedented quantified hemodynamic readings and distinguish treatment-specific from light fluence effects.

Interestingly, prolonged Taxotere therapy induced the formation of blood lakes (also known as vascular or hemorrhagic lakes) in a subset of KPL-4 tumors as revealed by MSOT imaging and cryosectioning, whereas no hemorrhage was detected in any of the vehicle-treated tumors. Detection of static blood pooling using optoacoustic imaging has also been demonstrated previously in a study employing dynamic contrast-enhanced ultrasound (DCEUS) and dual-wavelength optoacoustic tomography 59. The formation of blood lakes is associated with defects in the endothelial monolayer which lead to disruption of the vessel wall integrity (vessel leakiness) along with erythrocyte extravasation and pooling 60. These static pools of blood display poor perfusion with high hemoglobin content which produces a strong absorption signal with no apparent vascular structure in optoacoustic THb images 59. Furthermore, blood lakes in our MSOT images could be easily differentiated from areas of necrosis which typically display low or no hemoglobin signal. Importantly, the existence of blood lakes was confirmed by subsequent immunohistochemical analysis which showed an absence of CD31-positive blood vessels inside these hemorrhagic tumor areas. In addition, dynamic OE-eMSOT imaging revealed that the lakes were filled with de-oxygenated blood and showed little or no enhancement in sO_2_ upon application of the oxygen challenge. Notably, quantification of THb signal suggests that the increase in THb observed at the end time point of the imaging course in Taxotere-treated tumors (Figure 2A) was at least partially due to the formation of these blood pools, which occurred only towards the end of the imaging experiment. While we are not aware of previous reports of Taxotere-induced blood lake formation, our results are in general agreement with previous non-invasive *in vivo* imaging studies in animal tumor models 50, 61 and breast cancer patients 62 showing reduced vascular perfusion in response to taxane chemotherapy.

In our study, the *in vivo* findings on tumor oxygenation and blood volume were validated by rigorous analysis of histologic gold standards of vascularity (CD31) and tissue hypoxia (pimonidazole, CA9). On the basis of cryosection photography and CD31 staining, Taxotere-treated KPL-4 tumors were characterized by significantly lower microvascular density and deformed blood vessels in peripheral viable tumor regions along with the presence of large necrotic areas at their core. In addition, the overall vascular morphology revealed by CD31 labeling bears close resemblance to the *in vivo* distribution of THb in the corresponding co-registered MSOT images.

Crucially, a significantly higher mean hypoxic fraction (HF) was found in Taxotere-treated tumors compared with vehicle, based on immunohistochemical analysis of tumor cryosections stained against pimonidazole adducts. Accordingly, a significant anti-correlation between eMSOT-derived sO_2_ and pimonidazole-positive HF was found on a per-tumor-basis. Together, these two findings suggest that the reduction in whole tumor sO_2_ detected in Taxotere-treated tumors by eMSOT was accompanied by parallel increases in the fraction of severe tissue hypoxia (pO_2_ ≤ 10 mmHg 63) detectable by pimonidazole staining. It is important to acknowledge that, in our study, the effects of Taxotere treatment on tumor oxygenation were tested only in one mouse xenograft model. Thus, further studies in more clinically relevant patient-derived, transgenic as well as immunocompetent animal tumor models are required to test if the drop in tumor oxygenation is a generalized feature of solid tumors. Future research could also aim at evaluating eMSOT imaging in the clinical breast oncology setting.

This work may have direct translational implications for improving the therapeutic outcome of combined taxane treatment with therapeutic strategies that rely on oxygen availability. For instance, our results suggest that the therapeutic efficacy of taxane treatment could be potentially augmented by co-administration of hyperoxygenation therapy, as proposed recently 64. Finally, based on our findings it is anticipated that the oxygen lowering effects of Taxotere would result in synergistic antitumor activity with hypoxia-activated prodrugs (HAPs), as suggested by a previous study 65. Besides the immediate translational implications, the ability of eMSOT to map the heterogeneity in vascular function and oxygenation over the course of chemotherapy demonstrates its potential in preclinical drug assessment and evaluation of tumor therapeutic response.

## Conclusions

In summary, we have demonstrated that Taxotere treatment causes a significant reduction in oxygen saturation levels along with disruption of functional vascularization in the KPL-4 breast xenograft mouse model. Although the present study focused on the hemodynamic response of the TME to Taxotere therapy, our findings have broader implications for preclinical monitoring of therapeutic response in animal tumors. Furthermore, our results may have direct translational and clinical implications, for example in optimizing the timing of taxane administration with combined radiotherapy or other therapies that rely on oxygen availability. Altogether, this study demonstrates the potential of eMSOT imaging for longitudinal and dynamic monitoring of tumor microenvironmental responses to chemotherapeutic agents.

## Supplementary Material

Supplementary figures and movie legend.

Supplementary movie.

## Abbreviations

CA9: carbonic anhydrase 9
CD31: cluster of differentiation 31
DCEUS: dynamic contrast-enhanced ultrasound
DOT: diffuse optical tomography
eMSOT: eigenspectra multispectral optoacoustic tomography
ER: estrogen receptor
HAPs: hypoxia-activated prodrugs
HER2: human epidermal growth factor receptor 2
HF: hypoxic fraction
IHC: immunohistochemistry
MVD: microvessel density
MRI: magnetic resonance imaging
MSOT: multispectral optoacoustic tomography
NF: necrotic fraction
NACT: neoadjuvant chemotherapy
OE-eMSOT: oxygen-enhanced eigenspectra multispectral optoacoustic tomography
PBS: phosphate-buffered saline
PET: positron emission tomography
pO_2_: partial oxygen pressure
ROI: region of interest
RTV: relative tumor volume
sO_2_: oxygen saturation
THb: total hemoglobin concentration
TGI: tumor growth inhibition
TME: tumor microenvironment
