# Supplementary material for: Chemotherapeutic effects on breast tumor hemodynamics revealed by eigenspectra multispectral optoacoustic tomography (eMSOT)
Source: Theranostics. 2021 Jun 26;11(16):7813–28. doi: 10.7150/thno.56173 (PMC8315054; doi:10.7150/thno.56173)
Supplement: Supplementary file 1 — Supplementary figures and movie legend. [file thnov11p7813s1.pdf]

Supplementary Information for

Chemotherapeutic effects on breast tumor hemodynamics revealed by eigenspectra multispectral optoacoustic tomography (eMSOT)

Evangelos Liapis[1], Angelos Karlas[1,2], Uwe Klemm[1], and Vasilis Ntziachristos[1,2*]

[1] Helmholtz Zentrum München (GmbH), Neuherberg, Germany, Institute of Biological and Medical Imaging. [2] Technical University of Munich, Germany, School of Medicine, Center for Translational Cancer Research (TranslaTUM), Chair of Biological Imaging.

*Correspondence to: Vasilis Ntziachristos, Helmholtz Zentrum München, Institute of Biological and Medical Imaging Building 56 Ingolstädter Landstr. 1 D-85764, Neuherberg, Germany.

Phone: +49 89 3187-3852. E-mail: bioimaging.translatum@tum.de

Movie S1. Oxygen-enhanced eMSOT (OE-eMSOT) imaging reveals diminished oxygenation response in Taxotere-treated KPL-4 tumors.

Figure S1. Quantification of Hypoxic Fraction in KPL-4 tumors.

Figure S2. Individual KPL-4 tumor growth kinetics.

Figure S3. Longitudinal monitoring of spatiotemporal changes in blood volume and oxygenation of individual KPL-4 tumors using static MSOT/eMSOT imaging.

Figure S4. Longitudinal assessment of changes in blood volume and oxygenation of KPL-4 tumors in response to Taxotere treatment using static MSOT/eMSOT imaging.

Figure S5. Individual sO_2 kinetic curves for KPL-4 whole tumor, rim and core tumor subregions in vehicle (saline) and Taxotere-treated mice subjected to an oxygen challenge.

Figure S6. Cryoslice block imaging of frozen KPL-4 tumors.

Figure S7. Pimonidazole and CA9 display concordant staining patterns.

Movie S1. Oxygen-enhanced eMSOT (OE-eMSOT) imaging reveals diminished oxygenation response in Taxotere-treated KPL-4 tumors. Time-lapse video (10 fps) showing successively acquired eMSOT images of single central optoacoustic tumor cross-sections from representative vehicle (3 left panels) and Taxotere (5 right panels) treated mice. 9 eMSOT frames were obtained during medical air (21% O_2, grey arrow) and 11 frames under pure oxygen (100% O_2, green arrow) breathing conditions. A notable increase in tumor rim sO_2 is evident in vehicle tumors shortly after the exchange in breathing gas (4:30 min). A markedly reduced oxygenation response at both the rim and core subregions was observed in tumors from Taxotere-treated mice following the switch in breathing gas. Scale bars; 2 mm.

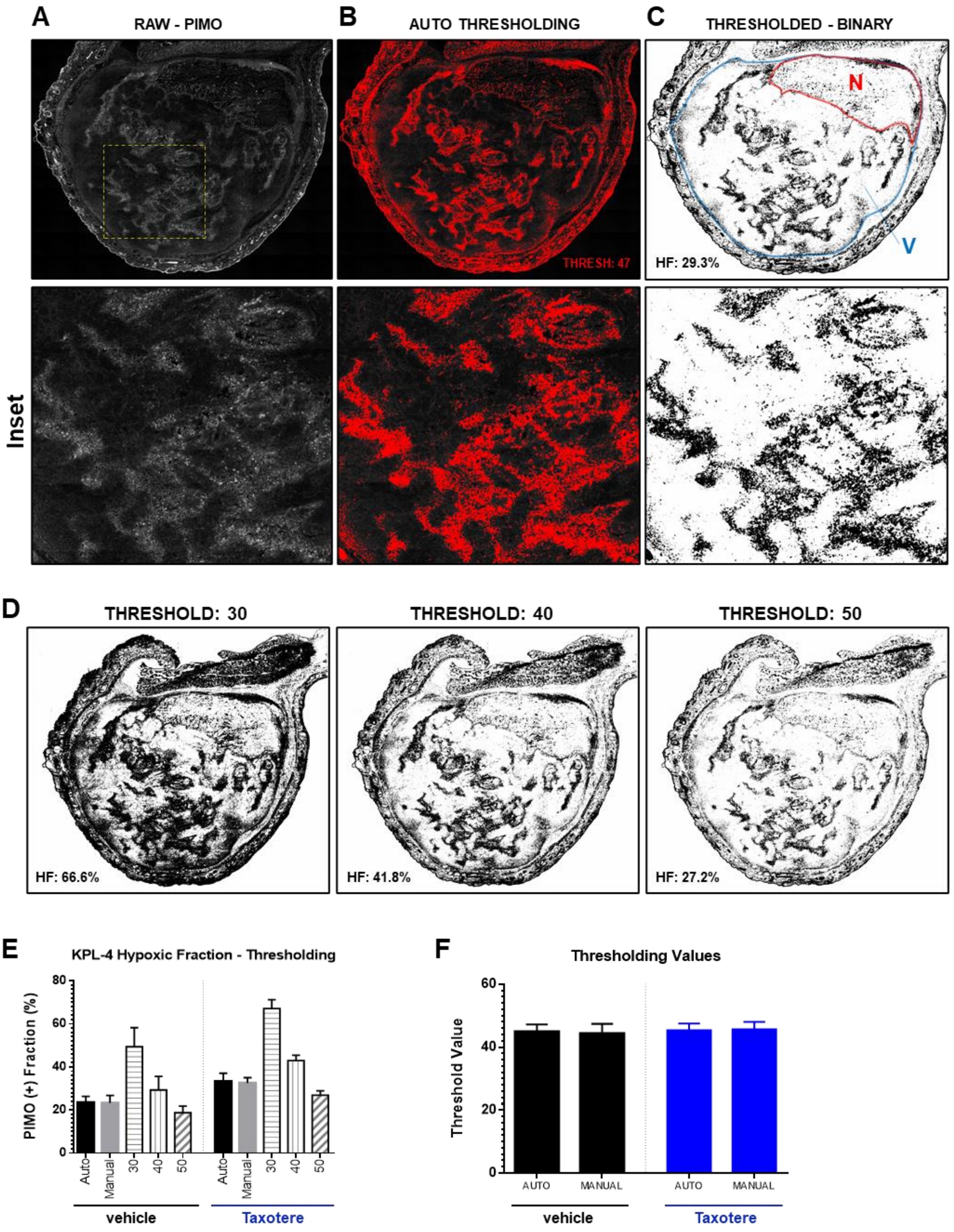
A
RAW - PIMO
B
AUTO THRESHOLDING
THRESH: 47
C
THRESHOLDED - BINARY
N
V
HF: 29.3%
Inset
D
THRESHOLD: 30
THRESHOLD: 40
THRESHOLD: 50
HF: 66.6%
HF: 41.8%
HF: 27.2%
E
KPL-4 Hypoxic Fraction - Thresholding
PIMO (+) Fraction (%)
Auto
Manual
30
40
50
vehicle
Taxotere
F
Thresholding Values
Threshold Value
AUTO
MANUAL
vehicle
Taxotere

Figure S1. Quantification of Hypoxic Fraction in KPL-4 tumors. (A) Fully stitched grey scale (raw) image showing pimonidazole immunofluorescence distribution in a transversal section of a representative Taxotere-treated KPL-4 tumor. **(B)** Selection of pimonidazole-positive pixels (shown in red) upon application of Otsu's threshold clustering algorithm (Auto Thresholding) which assigns global threshold values from grey-level histograms (see Methods). In this particular example, a global threshold value of 47 was computed by the algorithm. **(C)** Binarized, thresholded image. The hypoxic fraction (HF) was calculated as the percentage of pimonidazole-positive fluorescent area divided by the total viable (V) tumor tissue area, with the necrotic (N) areas excluded. In this particular case, the HF was 29.3%. Insets are magnified views of the yellow-dotted rectangular region shown in (A). **(D)** Binarized images of the same tumor shown in (A) following thresholding using 3 pre-defined selected threshold values (30, 40, 50). As can be seen in the binarized images, the optimal thresholding range was between 40 and 50, while values below 40 resulted in clear overestimation of HF **(E)** Bar graph showing quantification of HF using the entire tumor data sets from vehicle and Taxotere groups following Auto, Manual and 3-grader thresholding. Manual thresholding was performed by visual comparison of raw grey scale pimonidazole images and resulting binary thresholded images. As shown in the bar graph, manual determination of thresholding values resulted in similar estimates of HF to those obtained by Automatic Thresholding. **(F)** Mean threshold values calculated by Auto and Manual Thresholding for the entire vehicle and Taxotere-treated tumor datasets. The mean threshold values calculated for each group were: $Vehicle_{Auto}$ = 44.9 / $Vehicle_{Manual}$ = 45.1 / $Taxotere_{Auto}$ = 45.5 / $Taxotere_{Manual}$ = 45.7. Error bars in (E) and (F) represent the SD of the data. Only the Automatic Thresholding method was used for computation of HF in the results presented in Figure 4 in the main text.

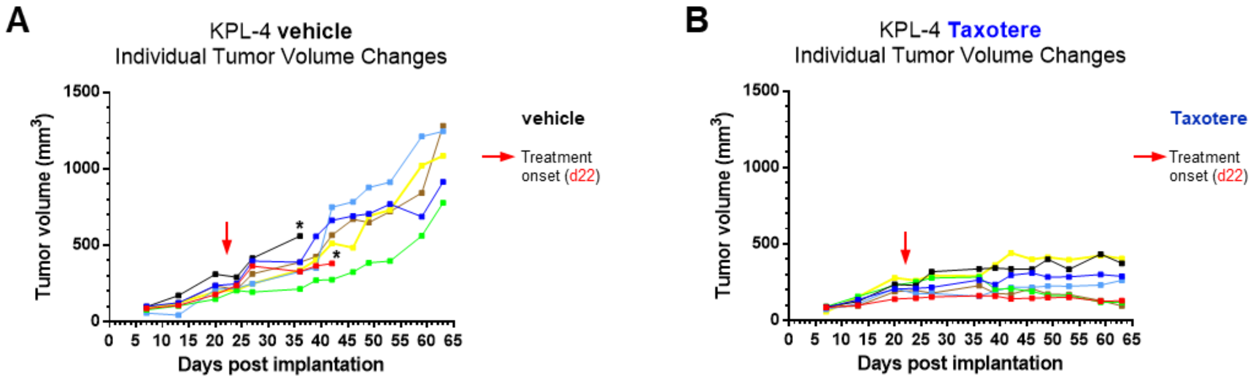

Figure S2. Individual KPL-4 tumor growth kinetics. Graphical plots showing changes in volume of individual KPL-4 tumors from **(A)** vehicle and **(B)** Taxotere-treated mice. Treatment with vehicle or Taxotere was initiated on d22 post implantation (red arrows). All 7 KPL-4 tumor-bearing mice responded to Taxotere treatment, although tumor growth inhibition varied between individual mice (B). *****2 vehicle-treated mice (A, black and red lines) were sacrificed at earlier time points (d36 and d42 post implantation) due to development of tumor ulceration (see Methods).

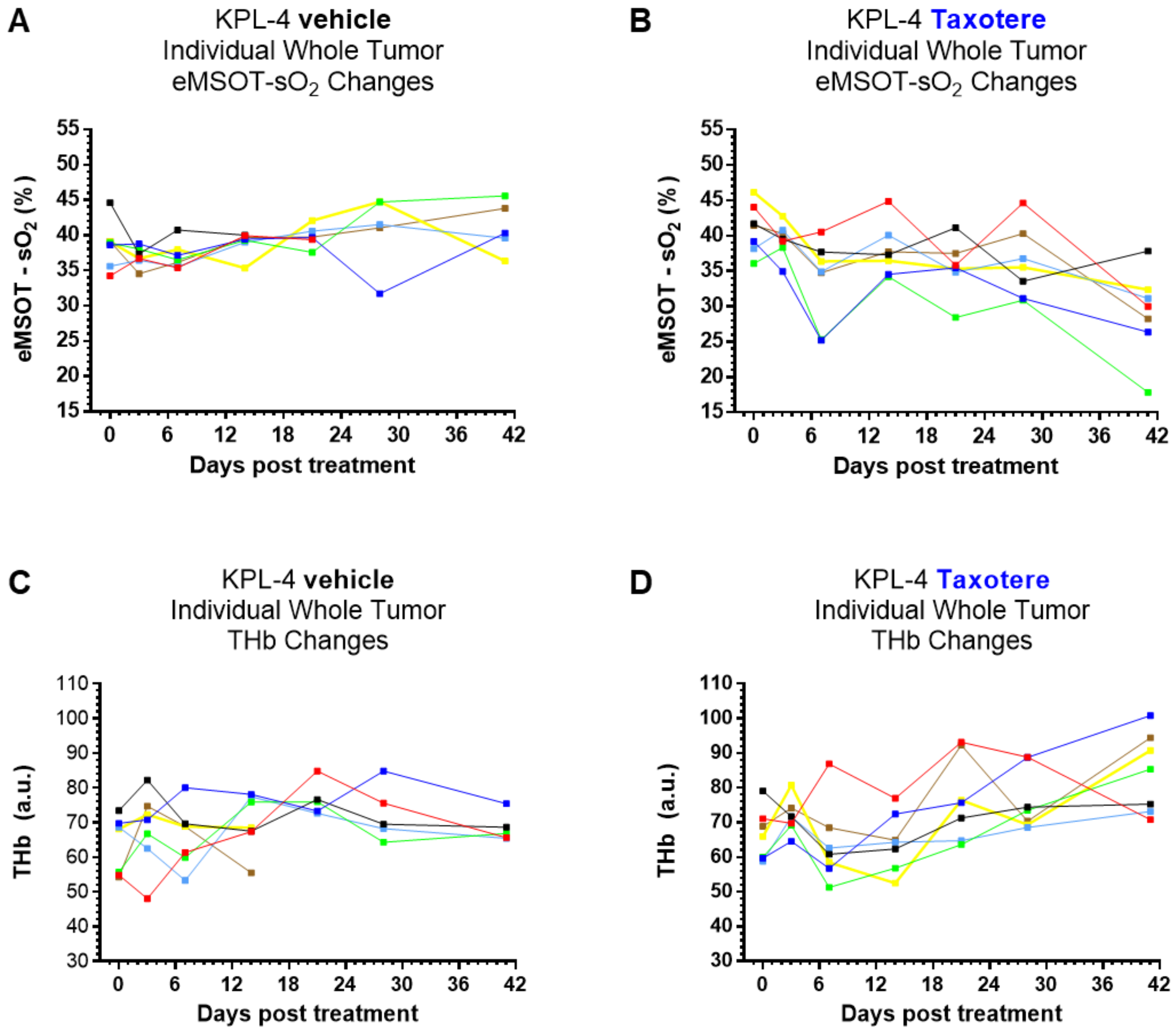

Figure S3. Longitudinal monitoring of spatiotemporal changes in blood volume and oxygenation of individual KPL-4 tumors using static MSOT/eMSOT imaging. (A-B) Graphical plots showing whole tumor sO_2 changes in individual KPL-4 tumors from **(A)** vehicle and **(B)** Taxotere-treated mice. **(C-D)** Individual whole tumor THb kinetic curves from **(C)** vehicle and **(D)** Taxotere-treated mice.

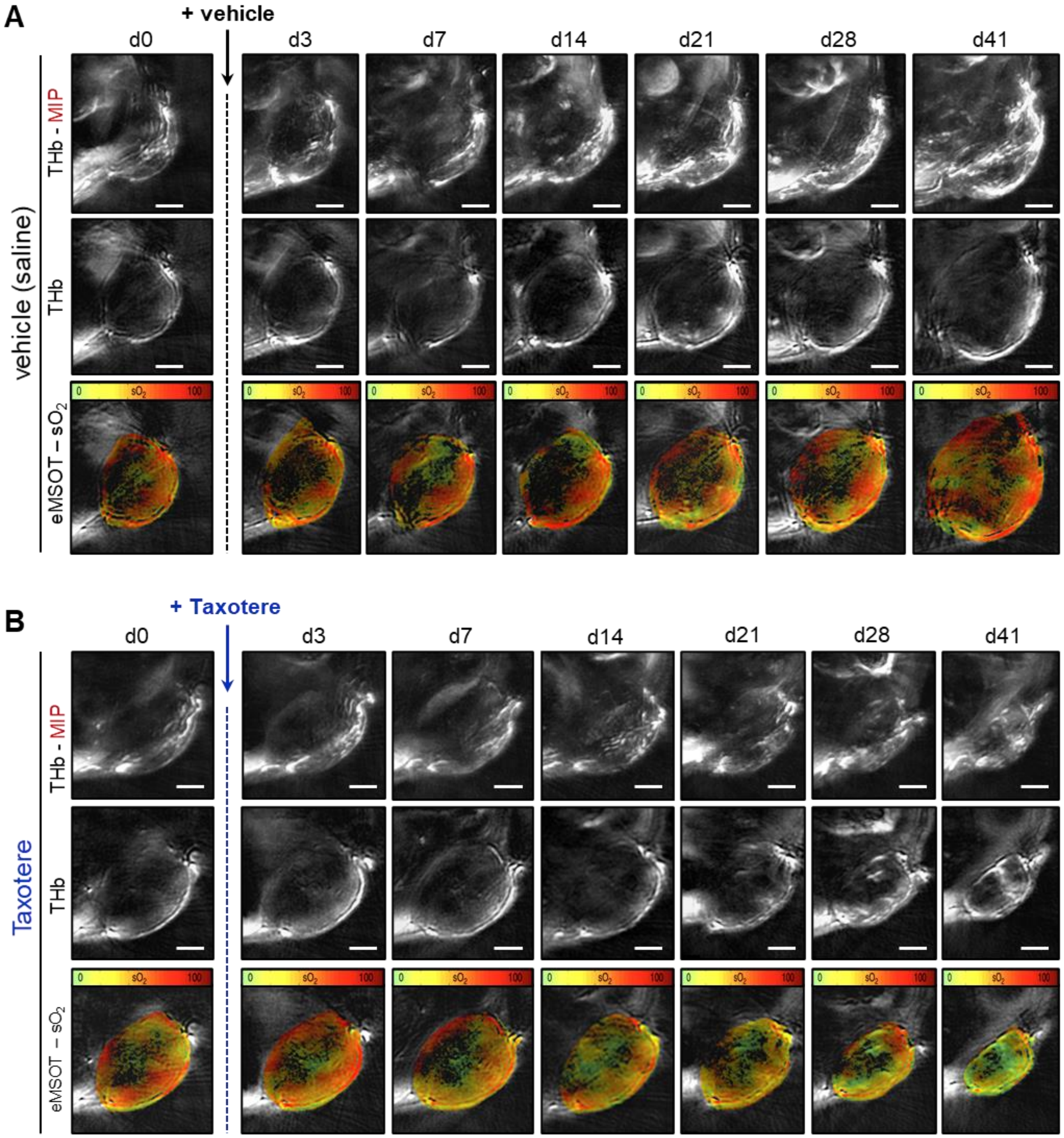

Figure S4. Longitudinal assessment of changes in blood volume and oxygenation of KPL-4 tumors in response to Taxotere treatment using static MSOT/eMSOT imaging. Tumor THb and sO_2 content visualized by 7 serial eMSOT images of single, representative tumors from **(A)** vehicle and **(B)** Taxotere-treated groups. Middle panels: central optoacoustic cross-sections (800 nm, isosbestic point) showing THb distribution. Upper panels: THb maximum intensity projections (THb-MIP). Lower panels: pseudocolorized eMSOT maps of tumor oxygen saturation overlaid on corresponding anatomical images. Color scale bars at the top of eMSOT images indicate sO_2 levels ranging from 0% (green) to 100% (red). Scale bars; 2 mm.

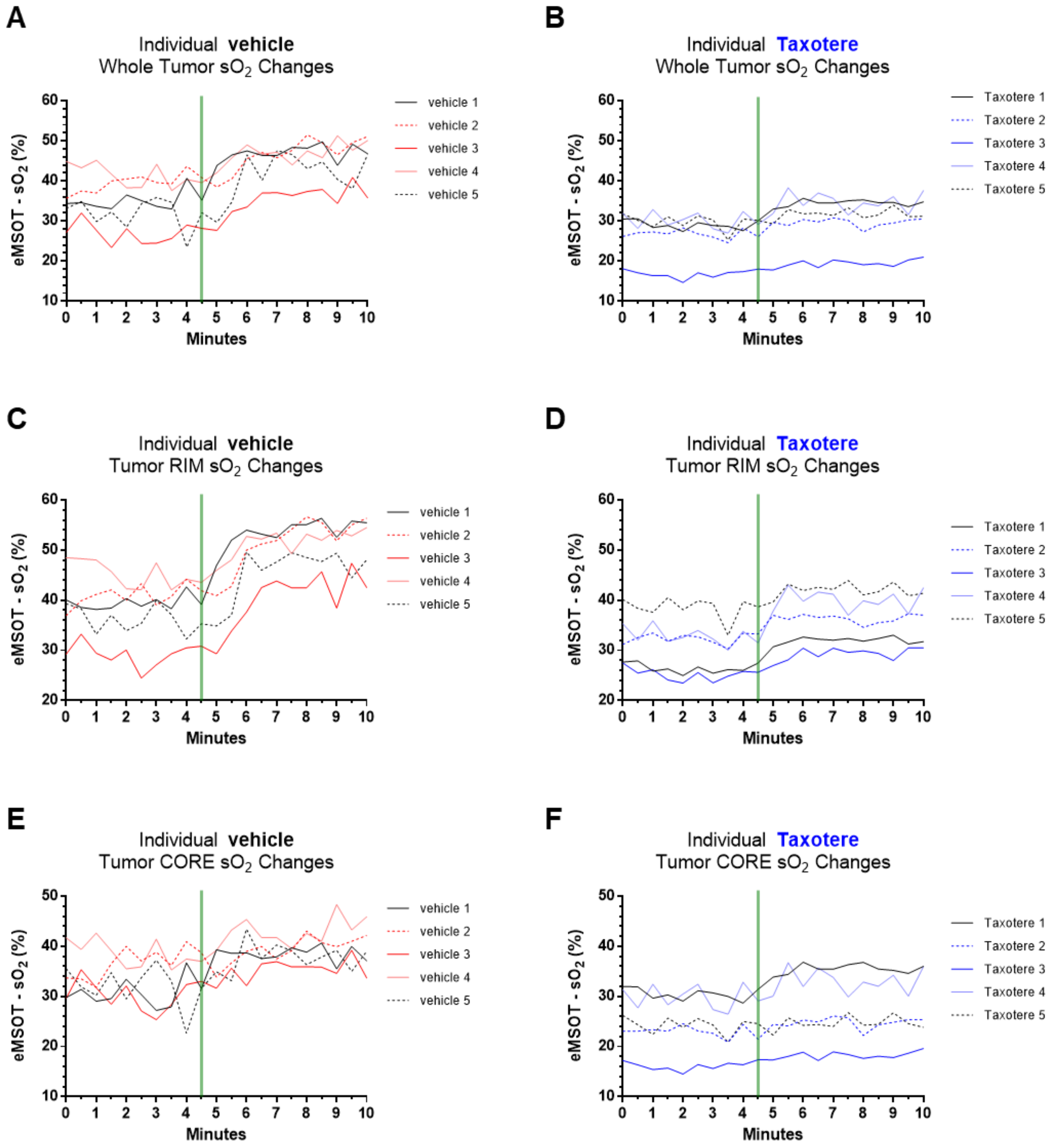

Figure S5. Individual sO_2 kinetic curves for KPL-4 whole tumor, rim and core tumor subregions in vehicle (saline) and Taxotere-treated mice subjected to an oxygen challenge. **(A)** Mean whole tumor sO_2 changes in individual tumors of vehicle treated mice. **(B)** Mean whole tumor sO_2 changes in individual tumors of Taxotere-treated mice. **(C)** Mean tumor rim sO_2 changes in individual tumors of vehicle treated mice. **(D)** Mean tumor rim sO_2 changes in individual tumors of Taxotere-treated mice. **(E)** Mean tumor core sO_2 changes in individual tumors of vehicle treated mice. **(F)** Mean tumor core sO_2 changes in individual tumors of Taxotere-treated mice. (n = 5 mice per treatment group). The switch from 21% to 100% O_2 is denoted by a vertical green line.

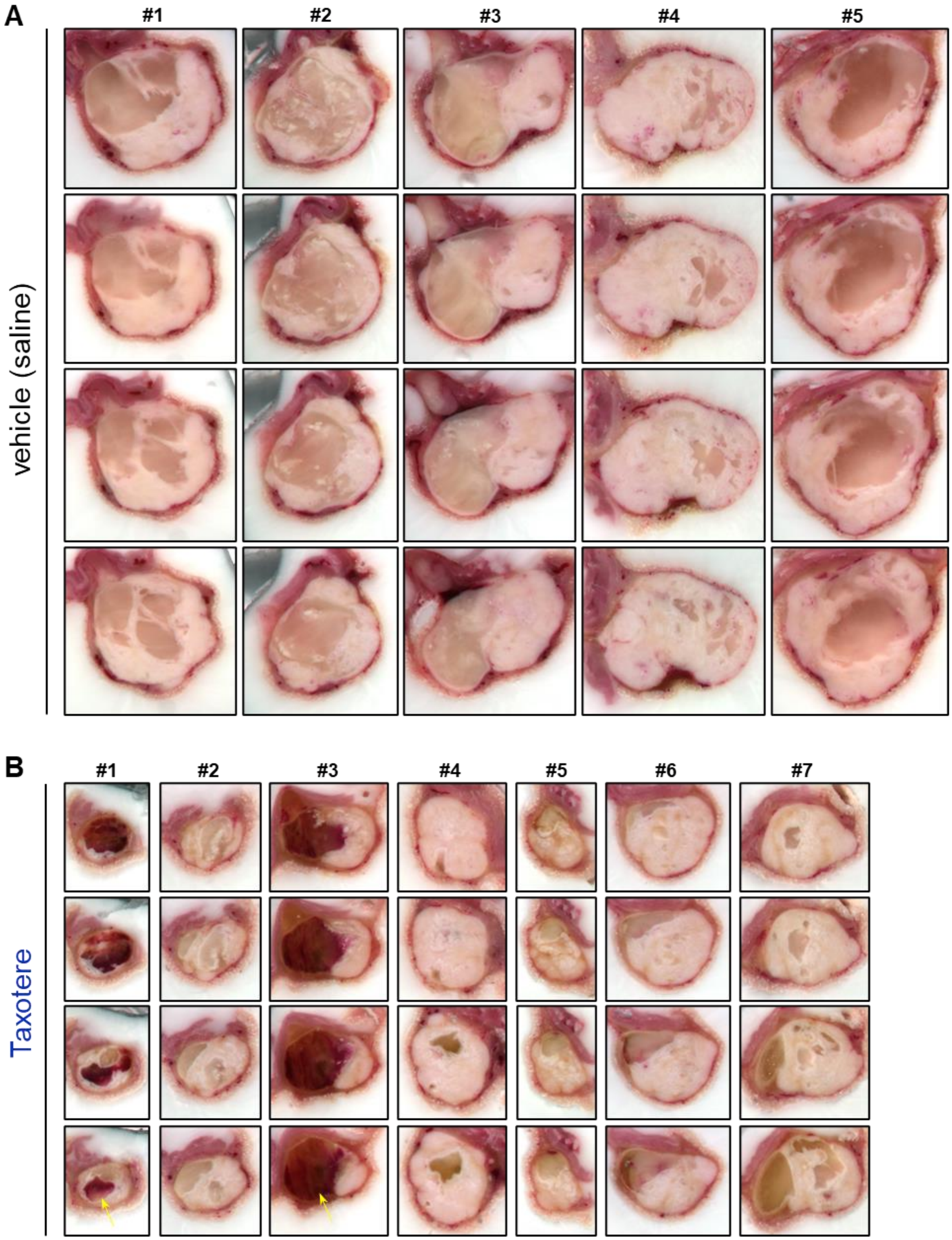

Figure S6. Cryoslice block imaging of frozen KPL-4 tumors. True color photographs of serial transversal KPL-4 tumor cryosections from **(A)** vehicle and **(B)** Taxotere-treated mice. Tumor sections are spaced by 0.5 mm. Yellow arrows in Taxotere-treated tumors #1 and #3 in (B) denote the presence of hemorrhagic blood lakes.

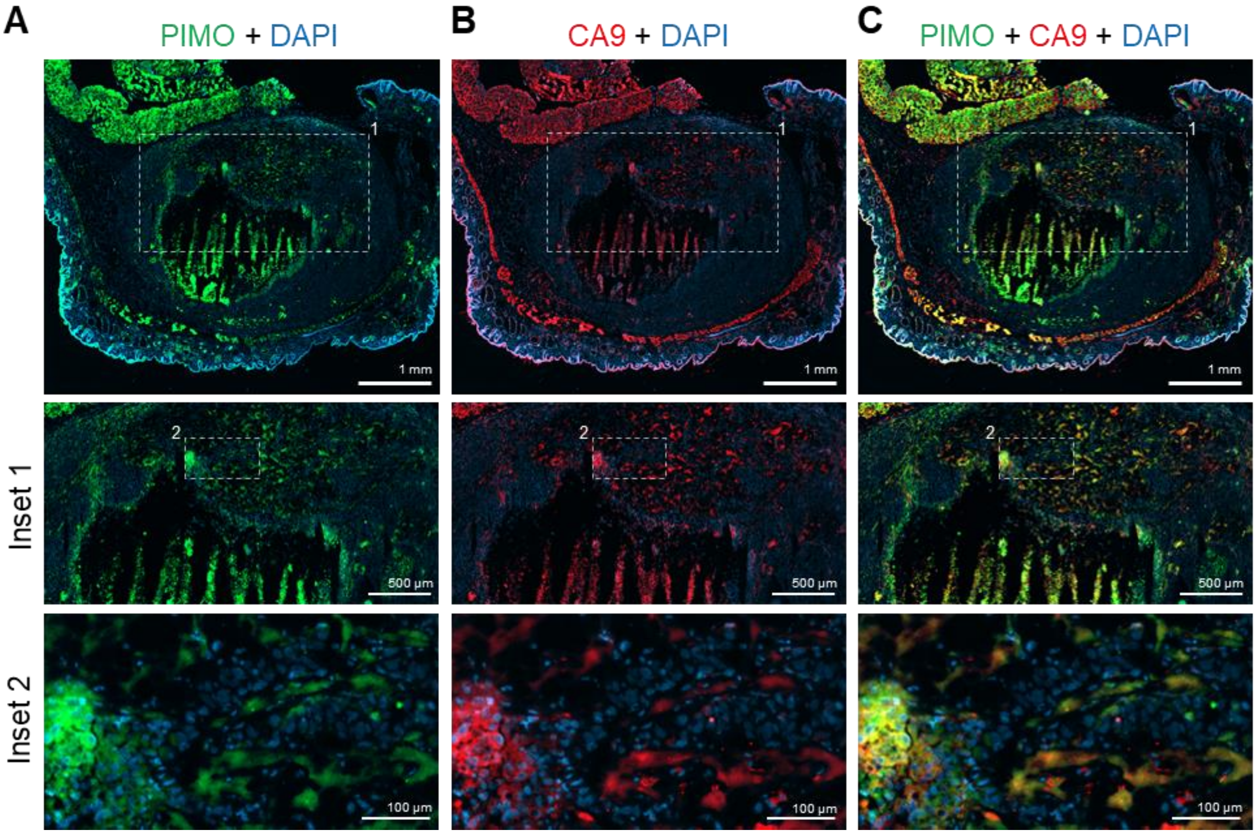

Figure S7. Pimonidazole and CA9 display concordant staining patterns. Fluorescence microscopic images of a Taxotere-treated KPL-4 tumor showing co-localization and hypoxia IHC staining patterns produced by **(A)** Pimonidazole (PIMO, green), **(B)** Carbonic Anhydrase (CA9, red), **(C)** Pimonidazole and CA9 merge. Images in (A-C) are counterstained with DAPI (blue). Insets 1-2 are successive magnifications of the white-dotted rectangles. Both hypoxia markers demonstrate perinecrotic staining as well as intense immunoreactivity against apoptotic/necrotic tumor cells localized inside the hemorrhagic blood lake at the centre of the tumor (see also Figure 5). Note the strong co-localization of pimonidazole and CA9 fluorescence in insets 1-2 of panel (C).
